# Exploited application of sulfate-reducing bacteria for concomitant treatment of metallic and non-metallic wastes: a mini review

**DOI:** 10.1007/s13205-016-0437-3

**Published:** 2016-06-03

**Authors:** Ali Hussain, Ali Hasan, Arshad Javid, Javed Iqbal Qazi

**Affiliations:** 1 Department of Wildlife & Ecology, University of Veterinary & Animal Sciences, Lahore, Pakistan; 2 Department of Zoology, University of the Punjab, Lahore, Pakistan

**Keywords:** Agro-industrial wastes, Beneficial microorganisms, Bioprecipitation, Economical bioremediation, Sulfate-reducing bacteria, Toxic metals

## Abstract

A variety of multidimensional anthropogenic activities, especially of industrial level, are contaminating our aquatic and terrestrial environments with a variety of metallic and non-metallic pollutants. The metallic and non-metallic pollutants addressed specifically in this review are heavy metals and various compound forms of sulfates, respectively. Direct and indirect deleterious effects of the both types of pollutants to all forms of life are well-known. The treatment of such pollutants is therefore much necessary before their final discharge into the environment. This review summarizes the productive utility of sulfate-reducing bacteria (SRB) for economical and concomitant treatment of the above mentioned wastes. Utilization of agro-industrial wastes and some environmental contaminants including hydrocarbons, as economical growth substrates for SRB, is also suggested and proved efficient in this review. Mechanistically, SRB will utilize sulfates as their terminal electron acceptors during respiration while utilizing agro-industrial and/or hydrocarbon wastes as electron donors/carbon sources and generate H_2_S. The biogenic H_2_S will then react vigorously with dissolved metals present in the wastewaters thus forming metal sulfide. The metal sulfide being water insoluble and heavier than water will settle down in the water as precipitates. In this way, three types of pollutants i.e., metals, sulfates and agro-industrial and/or hydrocarbon wastes will be treated simultaneously.

## Introduction

No doubt chemical as well as biotechnological industrial units supply us with a number of inevitable products. But pollution from industries cannot be ignored in addition to their usefulness. It is a nuisance causing the degradation of the environment by affecting the air, water and soil (Govindarajalu 2003). Industrial wastes and emissions contain toxic and hazardous substances, of which mostly are detrimental to human health as well as to the environment. Human health and environmental quality are being affected negatively by the perpetual production of industrial wastes (Adebisi and Fayemiwo 2011). In the context of the environmental pollution and its impact on health and global climatic change, the present time necessitates the importance of environmental remediation. This review emphasizes the importance of an economical bioremediation strategy, especially for developing countries like Pakistan that cannot afford much budget in the protection of their local environments. This notion is exemplified in this review by the synchronous bioremediation of three categories of pollutants, i.e., metals, sulfates and agro-industrial and/or hydrocarbon wastes originating from different industries. Sulfate-reducing bacteria (SRB) are advocated as remedial agents. A variety of organic wastes produced in agricultural lands like sugarcane bagasse, rice and wheat straw, animal manure, etc. are described as economical growth substrates in addition to some hydrocarbon contaminants, for the propagation of SRB. The proposed model is likely to solve the problems of metals and sulfate toxicity, as well as to improve the concerned soil and water habitats’ biology. This review falls into following subtopics:

## Metal pollutants of industrial origin and their detrimental health effects

Heavy metal pollution is becoming a significant concern in many countries because of its being non-biodegradable, persistent and thus bioaccumulative and continuous generation nature (Armitage et al. 2007; Sakan et al. 2009; Wang et al. 2013). Besides the dominant source of industrial origin, sewage water may also contain significant amounts of heavy metals such as zinc, iron, copper, manganese, lead, cadmium, chromium, nickel and cobalt, etc. (Idris et al. 2007; Malla et al. 2007; Zhang et al. 2016a). It is well known that all types of metals including radioactive ones are transferred to animals and human beings through food chains and exert harmful effects (Gall et al. 2015; Meena et al. 2016). According to WHO (1984) metals of the most immediate concern are aluminium, chromium, manganese, iron, cobalt, nickel, copper, zinc, cadmium, mercury and lead. Health effects of some commoner encountering heavy metals are given in the Table 1.

**Table 1:** Health effects of most commonly encountering heavy metals and their industrial sources of generation

Metal	Generation sources	Principal health hazards
Aluminium	Aluminium alloys’ production, packaging units, pharmaceutical industries	Aerial occupational exposure may produce lung fibrosis in humans In uremic patients, osteomalacia can occur due to aluminium in dialysis fluid May alter intestinal functions and metabolism of calcium in several organ systems
Cadmium	Alloys’ production, automotive and air craft industries, electroplating/galvanizing, metallurgical processing, mining, nickel–cadmium battery manufacturing industries, paint industries, plastic industries, textile printing	Affects the activity of alcohol dehydrogenase, arylsulfatase, delta-aminolevulinic acid dehydratase, delta-aminolevulinic acid synthetase, lipoamide dehydrogenase, pyruvate decarboxylase and pyruvate dehydrogenase Ingestion may result in disturbances in the gastrointestinal tract, vomiting, proteinuria, osteomalacia, liver dysfunction, kidney dysfunction/damage manifested by anemia and hypertension Long term low-level exposure leads to chronic obstructive pulmonary and renal tubular diseases and emphysema
Chromium	Cement manufacturing, chemical and refractory processing, chrome-plating, combustion of fossil fuels, ferrochrome production, metal-finishing industries, ore refineries, tanneries, textile plants	Low-level chronic exposure leads to kidney damage while occupational exposure may leads to asthma as well as cancer of the respiratory tract especially in the chrome production and chrome pigment industries May cause allergic dermatitis in humans
Cobalt	Cemented tungsten carbide industry, high temperature alloys’ manufacturing, paint industry	Exposure to low concentrations (0.002 to 0.01 mg/m^3^) causes respiratory irritation while to higher concentrations (0.1 mg/m^3^ or higher) can lead to “hard metal” pneumoconiosis Ingestion in excessive amounts can cause erythropiotic effects and cardiomyopathy Intravenous administration can cause deafness due to nerve damage, flushing of the face, giddiness, increased blood pressure, slowed respiration and tinnitus
Copper	Copper mining, metal fumes from smelting operations, welding	Excessive accumulation leads to Wilson’s disease Higher doses can cause anaemia, liver and kidney damage and irritation in stomach and intestine Ingestion of large amounts of copper sulfate may lead to hepatic necrosis and death
Iron	Hematite mining industries, metal industries, welding	Inhalation of iron oxide fumes or dust may leads to deposition of iron particles in lungs which produces an X-ray appearance like silicosis
Lead	Combustion of lead containing industrial emissions, glass polishing, hand loading of ammunition, jewelry making, lead-glazed pottery, painting, plastic industry, rubber industry, stained glass crafting	Deleterious effects include abdominal cramps, anorexia, insomnia, muscle aches, nausea, serious injuries to brain and kidneys, weakness of joints and weight loss It can pass the placental barriers and may reach the fetus resulting in miscarriages, abortions and still births In severe cases coma and death may occur
Manganese	Iron industry, welding	Chronic poisoning leads to a neuropsychiatric disorder characterized by difficulty in walking, irritability, speech disturbances and compulsive behaviour which may include fighting, running and singing
Mercury	Chlor-alkali industry, extraction of gold, in dentistry as amalgam tooth filling, paper industry, pulp manufacturing industry, smelting operations	Associated with kidney damage and its chronic poisoning may cause anemia, excessive irritation of tissues, gingivitis, loss of appetite, nutritional disturbances and salivation Inhalation of vapours at extremely high concentrations may lead to an acute, corrosive bronchitis and interstitial pneumonitis
Nickel	Combustion of fossil fuels, electroplating, fumes from alloys used in welding and brazing, metal plating industries, nickel mining, nickel-refining industries	Acts as a respiratory tract carcinogen
Zinc	Coal and waste combustion, mining, steel processing	Acute zinc toxicity leads to gastrointestinal distress and diarrhoea while inhalation of freshly formed fumes of zinc may cause metal fume fever

Literature adapted from these authors’ publications (Goyer and Clarkson 2001; Landis and Yu 2004; Scragg 2006; Becker et al. 2010)

## Sulfates containing industrial effluents and their deleterious effects

Among non-metal pollutants, various compound forms of sulfates deserve special attention. Continuous intrusion, especially from the wastes of industrial origins, of which effluents from edible oil production plants, food processing industries, paper mills, petroleum refineries, potato starch factories, pulp manufacturing industries, solid waste processing plants, tanneries and textile wastewaters make presence of different sulfur species in soils and waters at varying levels (Boshoff et al. 2004; Vaiopoulou et al. 2005; Huang et al. 2006). In ecologically viable locations, such pollutants are recycled by the microbes of sulfur cycle (Madsen 2008). However, in the situations where its presence behaves as a pollutant, many deteriorative processes like acidogenesis, corrosion of metals and H_2_S altered toxicological effects occurs (Lin and Hsiu 1997; Muyzer and Stams 2008; Lim et al. 2016; Zhao et al. 2016). In addition, human health is being affected negatively due to an exposure to sulfates. The most commonly encountering adverse health effects in human beings include acute renal failure, coma, confusion, cough, dyspnea, hepatotoxicity, increase in hippocampus superoxide dismutase (SOD), catalase (CAT) and glutathione peroxidase (GPx) activities, loss of consciousness, late sequelae of interstitial fibrosis, metabolic acidosis, myocardial necrosis, prolonged apnea, pulmonary edema, seizures, severe intravascular hemolysis, severe neurological impairment and shocks (Duong et al. 2001; Mbaye et al. 2003; Christia-Lotter et al. 2006; Kucukatay et al. 2007; Mortazavi and Jafari-Javid 2009).

## Remediation of metals and sulfates: scope and types

Treatment of metals and sulfates from industrial effluents is very much necessary before discharging them to the environment. There are a number of physicochemical treatment methods for the removal of metal ions from aqueous solutions. These include mainly electrodialysis, reduction, reverse osmosis, solvent extraction (Zhang et al. 1998) adsorption (Aguado et al. 2009) coagulation (El-Samrani et al. 2008) electrochemical precipitation (Chen and Lim 2005) filtration (Fatin-Rouge et al. 2006) and ion exchange, etc. (Dizge et al. 2009). However, such efforts require the use of energy and implication of more chemicals. In this way a pollutant can be recovered from the environment usually at the expense of adding more new chemical(s) to the scene. However, the chemical treatment methods have been declared environmentally non-compatible owing to their low treatment efficiency, complicated operation, high operational cost and the possible generation of secondary pollutions (Rocha et al. 2009; Ihsanullah et al. 2016). On the other hand, biological methods of the metals’ removal have gained importance for their better performance, low cost and environmentally compatible natures (Malik 2004; Okoh and Trejo-Hernandez 2006; Gillespie and Philp 2013). Bioremediation of metals’ containing effluents has experienced various shades including phytoremediation (Jadia and Fulekar 2009; Tauqeer et al. 2016) and biosorption (Hussein et al. 2004; Gupta et al. 2015; García et al. 2016). Although, both of the mentioned ways make the toxic metals generally non-available to the environment. But as regards their bioavailability, the plants and microorganisms may concentrate pollutants at various levels and being a component of food chains they may become the toxicants’ transferring agents in greater amounts at higher trophic levels (Peralta-Videaa et al. 2009).

The most recent and attractive approach for the treatment of metallic wastes is the precipitation of metal ions in the form of their respective sulfides. The counter reactant (hydrogen sulfide) of the metals needed for this process may be provided by the activity of SRB removing metals as well as sulfates concomitantly. However, the treatment of sulfates at larger scale has not been described by researchers still now.

## Biosulfidogenesis

Generation of hydrogen sulfide by microorganisms is known as biosulfidogenesis. It may occur via desulfhydration, sulfate reduction, sulfur respiration and sulfur disproportionation (inorganic ‘S’ fermentation). Due to its (1) proton consuming reaction (2) precipitating many metals and metalloids efficiently and (3) lowering the concentrations of sulfates (Jameson et al. 2010), the H_2_S has been employed for bioremediation of selected pollutant sites. Many different bacterial groups have the ability to reduce sulfate, thiosulfate, elemental sulfur and even can break down the sulfur containing amino acids in proteins to produce sulfide (Magot et al. 1997). However, SRB are the most widely studied biosulfidogens having the potential of remediating metal-rich contaminated wastewaters (Koschorreck 2008).

## Ecology and biotechnology of SRB

SRB make morphologically and physiologically a diverse group of obligatory anaerobes which share the ability to dissimilate sulfate to sulfide while oxidizing various growth substrates (Willis et al. 1997). These prokaryotic microorganisms are much versatile in their metabolism as well as in the environmental conditions in which they thrive and particularly make their importance in specific ecosystems such as acid mine drainages, cyanobacterial microbial mats, deep-sea hydrothermal vents, hypersaline microbial mats, marine and freshwater sediments, methane zone of marine sediments, oil fields’ environments, polluted environments such as anaerobic purification plants, rhizosphere of plants and rice fields (Fauque 1995; Dhillon et al. 2003; Rabus et al. 2006; Foti et al. 2007; Leloup et al. 2007; Ollivier et al. 2007; Muyzer and Stams 2008; Hussain and Qazi 2016; Wissuwa et al. 2016). In the above mentioned ecosystems, SRB have to cope with drastic physicochemical conditions including high temperature and high pressure, etc. SRB may represent the first respiring microorganisms and contribute to the complete oxidation of organic matter. They also play a key role in the overall biogeochemistry of various environments where they inhabit by the production of sulfide and/or metal reduction. Due to their key role in the marine carbon and sulfur cycles, the significance of SRB in high and low-sulfate environments is highly appealing for understanding the factors that influence their distribution, population size and metabolic activities in the seabed.

## Diversity of SRB

In the last few decades, through the use of 16S rRNA or dsrAB (dissimilatory sulfite reductase) genes as molecular markers, many SRB species have been reported. The dsrAB gene fingerprinting methods such as t-RFLP, DGGE and gel-retardation analyses have been used for rapid determination of SRB diversity in different environments (Wagner et al. 2005; Geets et al. 2006). More than 220 species of 60 genera of SRB have been described still now. They belong to five divisions (phyla) within the bacteria that are the Deltaproteobacteria, Firmicutes, Nitrospira and two phyla represented by *Thermodesulfobium* *narugense* and *Thermodesulfobacterium*/*Thermodesulfatator* species) and two divisions within the archaea (the euryarchaeotal genus *Archaeoglobus* and the two crenarchaeotal genera *Thermocladium* and *Caldivirga*, affiliated with the Thermoproteales) (Mori et al. 2003; Rabus et al. 2006; Ollivier et al. 2007; Muyzer and Stams 2008; Leloup et al. 2009). Rabus and Strittmatter (2007) reported that the complete genome sequences of nine SRB have been deposited in public databases. These include *Archaeoglobu fulgidus* (Euryarchaeota), *Caldivirga maquilingensis* (Crenarchaeota), the Gram-positive *Desulfotomaculum reducens* (Firmicutes) and six Gram negative Deltaproteobacteria: *Desulfobacterium autotrophicum*, *Desulfovibrio vulgaris* Hildenborough, *Desulfovibrio vulgaris* subsp. *vulgaris* DP4, *Desulfovibrio desulfuricans* G20, *Desulfotalea psychrophila* and *Syntrophobacter fumaroxidans*. Owing to bioremedial potential, it is important to know the nutritional requirements of the sulfidogenic bacteria for both strengthening the remedial processes as well as widening their applications in this regard.

## Nutritional aspects of SRB

SRB may have an autotrophic, litho-autotrophic, or heterotrophic respiration-type of life under anaerobiosis. While their possible microaerophilic natures have also been reported (Fauque and Ollivier 2004). Heterotrophic SRB utilize organic compounds as substrates, while autotrophic use CO_2_ as the carbon source and obtain electrons from the oxidation of H_2_ (Lens and Kuennen 2001). The latest biochemical and microbiological studies suggest that SRB can utilize a wide variety of substrates as electron acceptors and donors (Rabus et al. 2006; Hussain and Qazi 2012, 2014; Hussain et al. 2014a, b). In addition to different sulfur species (sulfite, sulfate, thiosulfate and tetrathionate) various other organic and inorganic compounds serve as terminal electron acceptors for these bacteria (Fauque et al. 1991; Fauque 1995; Fauque and Ollivier 2004; Rabus et al. 2006; Muyzer and Stams 2008). More than one hundred different compounds including sugars (fructose, glucose, etc.), amino acids (alanine, glycine, serine, etc.), alcohols (methanol, ethanol, etc.), monocarboxylic acids (acetate, butyrate, propionate, etc.), dicarboxylic acids (fumarate, malate, succinate, etc.) and aromatic compounds (benzoate, phenol, etc.) serve as potential electron donors for SRB (Fauque et al. 1991; Rabus et al. 2006; Liamleam and Annachhatre 2007; Huang and Kao 2015; Stasik et al. 2015; Meckenstock et al. 2016). In general, SRB prefer low-molecular weight organic compounds as carbon and energy sources.

## Cultivation of SRB using various environmental contaminants as growth substrates

Dissimilatory sulfate reducers have been reported to utilize lactate as a preferred carbon source and thus most widely employed for cultivating DSRB at laboratory scale (Barnes 1998; El-Bayoumy et al. 1999). However, lactate is too much expensive for a large scale practice. Hydrogen gas can also be used as an energy source by some DSRB (Lens et al. 2003). Although hydrogen is a relatively inexpensive substrate, yet it cannot be considered an acceptable energy source because of engineering and safety measures on a commercial scale while ethanol has been reported as a cost-effective substrate (Huisman et al. 2006). Several different natural sources of organic materials such as animal manure, sugarcane bagasse, leaf mulch, molasses, mushroom compost, fruit wastes, sawdust, sewage sludge, vegetal compost, whey and wood chips have been described as electron donors and carbon sources for the cultivation of SRB (Annachhatre and Suktrakoolvait 2001; Costa and Duarte 2005; Coetser et al. 2006; Hussain and Qazi 2012, 2014; Hussain et al. 2014a, b). Researchers have also demonstrated tannery effluents and wastes from the wine industry for supporting growth of dissimilatory SRB to economize certain bioremediation strategies (Boshoff et al. 2004; Martins et al. 2009a). SRB can utilize a range of different other environmental contaminants such as petroleum hydrocarbon constituents (e.g. alkanes, benzene, ethylbenzene, polycyclic aromatic hydrocarbons, toluene, xylenes) or halogenated compounds directly as a source of carbon and energy (Fauque et al. 1991; Hao et al. 1996; Harms et al. 1999; Morasch et al. 2004; Huang and Kao 2015; Stasik et al. 2015; Meckenstock et al. 2016). Recent data on SRB report that they can grow on long-chain alkanes (Davidova et al. 2006; Kleindienst et al. 2014; Herath et al. 2016), alkenes (Grossi et al. 2007; Fullerton et al. 2013) and short-chain alkanes (Kniemeyer et al. 2007). The above mentioned metabolic diversity and versatility of SRB in terms of their potential of using the range of carbon and energy sources is highly promising for designing strategies addressing bioremediation of metals and sulfates.

## Applications of SRB

The exploitation of SRB for the treatment of industrial wastewaters is of great interest. A number of studies, based on the applications of SRB have been carried out for the treatment of simulated and real wastewaters contaminated with a range of pollutants. The latest advancement in the applications of SRB have shown that SRB are used to treat various environmental contaminants including metals (Hussain and Qazi 2016; Mothe et al. 2016; Zhang et al. 2016b), metalloids (Battaglia-Brunet et al. 2012; Altun et al. 2014; Sahinkaya et al. 2015) sulfates (Hussain and Qazi 2014, 2016; Hussain et al. 2014a), methane (Krukenberg et al. 2016), various non-methane hydrocarbons e.g. alkanes (Callaghan et al. 2012; Khelifi et al. 2014; Kleindienst et al. 2014; Herath et al. 2016) and alkenes (Fullerton et al. 2013), alicyclic hydrocarbons e.g. cyclohexane (Jaekel et al. 2015), aromatic hydrocarbons e.g. benzene (Huang and Kao 2015; Aüllo et al. 2016; Meckenstock et al. 2016), naphthalene (Kümmel et al. 2015; Meckenstock et al. 2016), phenanthrene (Sayara et al. 2015; Meckenstock et al. 2016), toluene (Huang and Kao 2015; Stasik et al. 2015; Aüllo et al. 2016), xylene (Huang and Kao 2015; Stasik et al. 2015), ethylbenzene (Stasik et al. 2015; Aüllo et al. 2016) and 2-methylnaphthalene (Folwell et al. 2015) and nitroaromatic compounds e.g. trinitrotoluene (Boopathy 2014; Mulla et al. 2014).

Almost all of the mentioned investigations were carried out at laboratory scale; however, no data are available on the commercial-scale applications of SRB. Only two patented technological applications, based on the microbially mediated sulfate reduction in bioreactor systems, have been developed and operated as pilot-, demonstration- and full-scale plants for the treatment of acidic wastewater from metal mines and related sites (Johnson 2000): Thiopaq^®^ by Paques, The Netherlands (Boonstra et al. 1999; Buisman et al. 2007) and BioSulphide^®^ by BioteQ, Canada (Rowley et al. 1997; Ashe et al. 2008). The most probable reason of poor applicability of SRB at commercial scale may be the uncontrolled generation of H_2_S exhaust. According to Martins et al. (2009b) additionally produced H_2_S (unreacted) easily escapes as a gas being some of it not accessible to the pollutants and thus the treatment of pollutants can never be quantitative. The escaped H_2_S my pose severe environmental impacts as well. This information necessitates optimization of sulfidogenesis and the wastes to be treated within tangibly designed bioreactors allowing maximum contact area and time for the H_2_S and waste(s) to react (Hussain and Qazi 2016). The H_2_S exhaust can also be controlled by making the entire remedial setup closed. Some sort of bio/technical control of unwanted (additionally produced) H_2_S is, therefore, recommended to make the commercial-utility of SRB feasible.

## Economical and concomitant treatment of metals and sulfates

Big cities of developing countries represent one of the major sources of water pollution. Wherein untreated domestic and industrial effluents are ultimately thrown into streams and rivers. The biota of the concerned lotic environments has been changing its composition rapidly. While the withstanding populations are being affected negatively in terms of their population density and biochemical alterations. Recalcitrant pollutants, especially metals are transferring through the food chains to humans. Untreated sewage effluents cause high BOD and COD levels tremendously and the resultant anaerobiosis may escalate the growth of sulfidogenic bacteria yielding H_2_S in selected locations. Such situations have contaminated the environment with increasing populations of harmful microorganisms and their byproducts.

Keeping in view the above mentioned facts, metal-tolerant sulfidogenic bacteria have been perceived an appealing condition for precipitating metals from effluents in the form of their sulfides (Gadd 2000; Hussain and Qazi 2016). SRB play an important role in metal sulfide immobilization in anaerobic environments that contain high concentrations of metals (Kaksonen et al. 2004; Van Roy et al. 2006). This remedy, however, requires the provision of physical factors and nutrients which can support the growth of relevant microbes as well as production of H_2_S. Domestic and industrial sewages rich in organic contents, themselves pollutants in the environment, may provide the nutritional requirements of sulfidogenic bacteria. Other nutritional requirements of sulfidogenic bacteria can also be accomplished from different agro-industrial wastes on low/no cost basis. Blending of suitable carbon-sources (electron donors) may support indigenous or inoculant microbial communities capable of reducing sulfates to sulfides by precipitating metal contaminants.

**Fig. 1:**
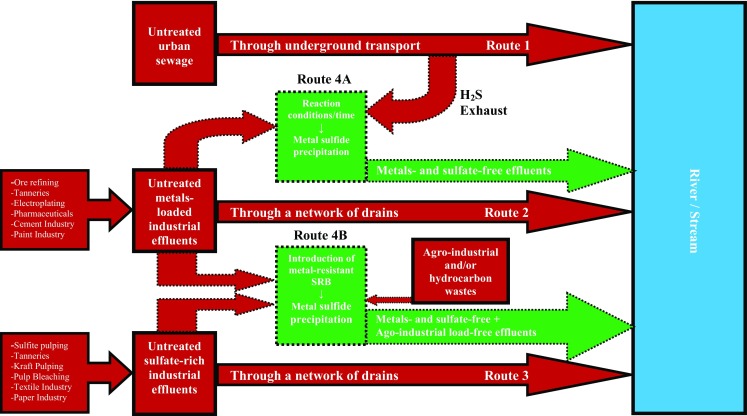
Routes 1, 2 and 3 represent the present situation of untreated sewage, metal and sulfate containing industrial effluents, respectively while routes 4A and 4B indicate two different possible bioremediating fates of metals and H_2_S and metals and other sulfate pollutants, respectively. In route 4B SRB growth is accomplished by agro-industrial and/or hydrocarbon wastes

The above discussed facts suffice to advocate detrimental effects of metals and sulfates as well as potential of suflidogenic bacteria for remediating these pollutants concomitantly. For this purpose, a biphasic model is proposed in Fig. 1 which also shows the routes of untreated effluents. The figure route 4A employs H_2_S exhaust, produced from sewage effluents by sulfidogenic bacteria under anaerobic conditions to precipitate metals from diverse industrial effluents. While the route 4B illustrates the importance of metal-resistant and heterotrphic sulfidogens for single-chambered bioremediation process development addressing the two categories of the pollutants concurrently.

## Concluding remarks

This review arrives at the conclusion that mixed industrial effluents, loaded with metals and sulfates, can be treated concomitantly using metal-resistant SRB. In addition, different types of agro-industrial and/or hydrocarbon wastes can be used as growth substrates for the efficient propagation of SRB. Infact, this approach leads to the treatment of three categories of pollutants i.e., metals, sulfates and agro-industrial/hydrocarbon wastes. Practical work on these lines at is likely to identify more suitable wastes which may resume the status of ingredients of a suitable medium for the cultivation of desired microorganisms capable of remediating the diverse pollutants. The authors of this review in addition to other researchers have also worked on different carbon sources for biological sulfate reduction. However, more work is required to identify the suitable SRB and the environmental wastes for their growth to meet the desired goal of economical bioremediation. In addition, exploitation of SRB on pilot and commercial scales is also necessarily required to further investigate the effectivess of closed and tangibly designed bioreactors and to improve the process(s) accordingly.
